# Directions and applications of CRISPR technology in livestock research

**DOI:** 10.21451/1984-3143-AR2018-0075

**Published:** 2018-08-17

**Authors:** IsmaeI Lamas-Toranzo, Priscila Ramos-Ibeas, Eva Pericuesta, Pablo Bermejo-Álvarez

**Affiliations:** 1 Department Reproducción Animal, INIA, 28040 Madrid, Spain.

## Abstract

The ablation (KO) or targeted insertion (KI) of specific genes or sequences has been essential to test their roles on a particular biological process. Unfortunately, such genome modifications have been largely limited to the mouse model, as the only way to achieve targeted mutagenesis in other mammals required from somatic cell nuclear transfer, a time- and resource-consuming technique. This difficulty has left research in livestock species largely devoided of KO and targeted KI models, crucial tools to uncover the molecular roots of any physiological or pathological process. Luckily, the eruption of site-specific endonucleases, and particularly CRISPR technology, has empowered farm animal scientists to consider projects that could not develop before. In this sense, the availability of genome modification in livestock species is meant to change the way research is performed on many fields, switching from descriptive and correlational approaches to experimental research. In this review we will provide some guidance about how the genome can be edited by CRISPR and the possible strategies to achieve KO or KI, paying special attention to an initially overlooked phenomenon: mosaicism. Mosaicism is produced when the zygote´s genome edition occurs after its DNA has replicated, and is characterized by the presence of more than two alleles in the same individual, an undesirable outcome when attempting direct KO generation. Finally, the possible applications on different fields of livestock research, such as reproduction or infectious diseases are discussed.

## Introduction

Genome modification has been crucial to understand the molecular root of physiological or pathological processes. The ablation (knock-out, KO) or insertion (knock-in, KI) of specific genes or sequences have allowed to unequivocally assess the role of a specific gene product on a particular process, to assess the spatial and temporal expression of a gene or to modify its expression pattern, among other applications. KO generation requires targeted mutagenesis (i.e., the modification of the genome at a specific locus), and targeted KI (i.e., the insertion of a sequence at a specific locus) is also preferred to random KI. Most experiments involving KO or KI models have been carried out in the only mammalian species where targeted genome modification was easily achievable: the laboratory mouse. In this sense, although non-targeted mutagenesis, achieved by different means such as such as pronuclear injection ( Hammer *et al*., 1985 ), transduction ( Chan *et al*., 1998 ) or mediated by intracytoplasmic sperm injection ( Shemesh *et al*., 2000 ) have been applied to farm animals, the only available method to achieve targeted mutagenesis, homologous recombination, was difficult to apply to livestock species.

Homologous recombination (HR) is a genome modification technique based on an homonymous DNA repair mechanism that can be directed to insert a given sequence in a specific genomic locus. The main drawback of this technique is that the proccess is extremely inefficient, resulting in insertion rates below 0.1% ( Brinster *et al*., 1989 ). This handicap can be bypassed by performing HR in Cell Cultures, where the few cells containing the intended modification after HR can be selected by introducing a selection cassette for resistance to a cytotoxic agent ( Doetschman *et al*., 1988 ). Once the genetic modification has been introduced into the cell genome, there are only two possible strategies to obtain a genetically modified animal. The first method to be developed was the use of genetically modified Embryonic Stem Cells (ESCs) for embryonic aggregation. This strategy generates chimeric animals partly composed of genetically modified cells derived from the ESCs. By this approach, if the genetically-modified ESCs-derived cells have formed germinal cells, the genetic modification could be transmitted to the offspring ( Evans *et al*., 1985 ). The main limitation of this strategy was that it could only be applied to mice, as truly pluripotent ESCs –hence able to derive into germinal cells- could not be obtained in other species.

The second approach to produce genetically modified offspring from genetically modified cells is to perform Somatic Cell Nuclear Transfer (SCNT). In this case, the genetically modified nucleus of a somatic cell (usually a fibroblast) is reprogrammed by the ooplasm of an enucleated oocyte, resulting in an individual entirely composed by cells containing the genetic modification ( Schnieke *et al*., 1997 ). This method allowed site-specific genome modifications in livestock species, but its application was highly restricted due to several technical limitations. SCNT is a technique difficult to master and very inefficient, resulting in less than 5% delivery rates ( Wilmut *et al*., 1997 ; Kato *et al*., 2000 ) and often yielding to developmental defects associated with deffective epigenetic reprogramming of the donor genome. Furthermore, the donor somatic cells used for HR are mortal, unlike ESCs, so they can senescence over the multiple passages required to perform the genetic modification, leading to the loss of the transgenic cellular line or in even lower embryo developmental rates following SCNT.

The technical constraints associated to targeted mutagenesis in farm animals have restricted the myriad of applications of genome modification in these species. Luckily, the advent of targeted mutagenesis techniques based on site-specific endonucleases has unleased the potential of genome editing in livestock species. Genome edited animals have been produced by different site-specific endonucleases such as Zinc-Finger Nucleases (ZFN) ( Geurts *et al.*, 2009 ; Whyte *et al.*, 2011 ), Transcription Activator-Like Effector Nucleases (TALEN) ( Tesson *et al.*, 2011 ; Carlson *et al.*, 2012 ) and Clustered Regularly Interspaced Short Palindromic Repeats (CRISPR) ( Shen *et al*., 2013 ; Wang *et al*., 2013 ), but due to the ease of use and flexibility, CRISPR has become the most popular method.

## Mutagenesis induction by CRISPR

CRISPR technology has its origin on an adaptive immune system from prokaryotes which retain memory of past viral exposures by storing short fragments of the viral DNA ( Mojica *et al*., 2005 ). Between the diverse CRISPR system existing in nature, several class II systems have been adapted for genome editing in eukaryotes ( Ran *et al*., 2015 ). The most commonly used system derives from the type II CRISPR system of the bacteria *Streptococcus pyogenes*, and it is composed by a Cas9 protein (CRISPR associated nuclease) and a sgRNA (single-guided RNA, which directs Cas9 to the target site, composed by 20 nucleotides followed by –NGG) ( Jinek *et al*., 2012 ).

**Figure 1:**
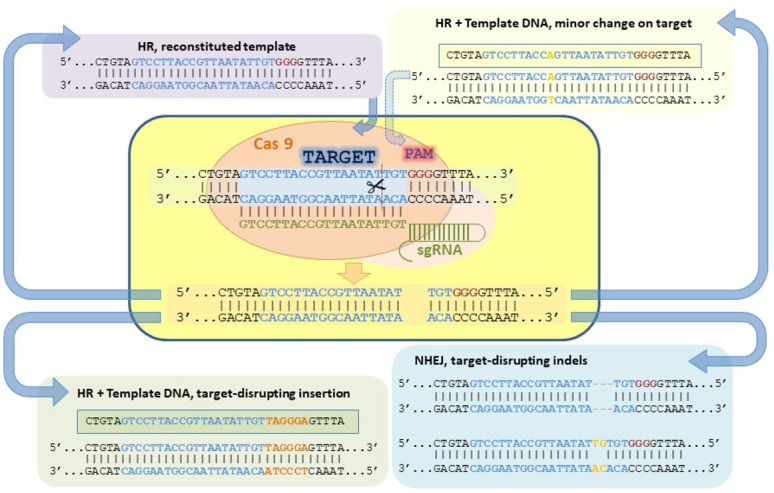
Dynamics of DSB repair by endogenous eukaryotic mechanisms (NHEJ or HR). Repairment by NHEJ often results in indels at the target site that impair CRISPR recognition. In contrast, repairment by HR reconstitutes the CRISPR target site unless a recombination template containing a target-disrupting insertion is provided. The reconstitution of the CRISPR target site leads to a new DSB at the repaired target unless CRISPR activity has ceased.

CRISPR, as other site-specific endonucleases, is able to find its particular target across the genome and induce a DNA double stranded break (DSB) at that locus. In this sense, CRISPR *per se* does not generate any mutation, the mutation is actually generated by the endogenous DSB repair mechanisms of the eukaryotic cell. Eukaryotic cells mainly repair DSB by one of two mechanisms: Non-Homologous End Joining (NHEJ) or Homologous Recombination (HR). The editing process is dynamic, as CRISPR remains active after one repair mechanism has fixed the DSB ( Figure 1 ). In this sense, if the repair mechanism has reconstituted the CRISPR target site or it has only slightly modified it, CRISPR will recognize the repaired site and generate a DSB again. The cycle will continue until CRISPR activity ceases or a modification in the target site impedes CRISPR recognition and thereby DSB generation. NHEJ is an error prone mechanism that often introduces or deletes bases (insertion/deletion, known as indel) at the DSB in the repair process ( Moore and Haber, 1996 ), thereby producing mutated sequences that are not recognized by CRISPR. In contrast, HR uses another DNA molecule as template ( Orr-Weaver *et al*., 1981 ) and thus, in the absence of any exogenous DNA, it reconstitutes CRISPR target site. Therefore, if CRISPR remains active after HR repair, it will reproduce the DSB on the repaired site. In contrast, if a template for homologous recombination able to modify CRISPR target site is provided ( Capecchi, 1989 ), this mechanism could be used to introduce DNA sequences at specific loci (KI).

## CRISPR for KO generation

**Figure 2:**
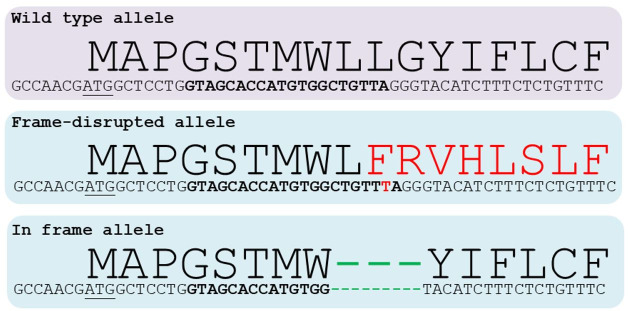
Examples of indels generated by CRISPR at the beginning of the coding region of rabbit *ZP4* gene. Wild-type, frame-disrupted and in frame alleles are shown. For each allele, aminoacid sequence is depicted in big letters that match the codons situated below, start codon (ATG) is underlined and CRISPR target site is marked in bold letters. On the frame-disrupted allele, a insertion of a single base (red T) disrupt the aminoacid sequence beyond that point. In contrast, a in frame indel consisting in a 9 bp deletion only eliminates 3 aminoacids, leaving the rest of the sequence unaltered.

The indels generated by NHEJ are the most common way to generate a KO by CRISPR. For this aim, CRISPR components are directly injected into a zygote, and CRISPR target site should be located at the beginning of the Open Reading Frame (ORF) of the target gene. On that region, if the indel generated is not multiple of three, it will originate a disruption of the ORF (frame-shift mutation), leading to a truncated and non-functional peptide (i.e., a KO allele). However, as indels are randomly generated, some will be multiple of three, resulting in the insertion or deletion of few aminoacids, but leading to a probably functional protein ( Figure 2 ). In other words, although virtually 100% gene editing efficiency can be achieved, 100% KO generation is statistically unachievable, as some of the indels generated will be multiples of 3 and thereby will not disrupt gene translation. In this context, genotyping strategy should be able to detect all indels (alleles) generated on a given individual, as solely individuals containing only frame-disrupting indels can be considered as KO.

A strategy to increase the percentage of KO out of edited embryos may be the use of multiple guides for the same gene ( Wang *et al*., 2015b ; Chuang *et al*., 2016 ; Wang *et al*., 2016a ; Wang *et al*., 2016b ; Vilarino *et al*., 2017 ). Multiple guides lead to multiple DSB that may result in either the deletion of a large fragment within them, which may include the start codon, or in the alteration of the ORF at different points. However, this strategy holds several drawbacks: 1) the indel generated on downstream DSB may reconstitute again the ORF disrupted by a first indel, resulting only in an alteration of the fragment between both DSBs, leading to a partially modified protein with unpredictable functionality, which contrasts with the neat and simple alleles generated with a single target; 2) for the same reason, the genotyping is more complicated and difficult to interpret; and 3) it increases the chances of offtarget.

As it will be discussed below, one of the main advantages of CRISPR over HR is that it allows the direct generation of KO individuals by direct injection of CRISPR components into a zygote. Due to the low efficiency of the technique, HR achieves monoallelic modifications in either ES or fibroblasts, resulting in heterozygote founders that need to be crossed to obtain a homozygous KO. The generation of a KO individual in one step (i.e. homozygous KO on F0 generation) is particularly useful to understand the role of specific genes during embryo development and it is extremely important to reduce the number of generations required to produce a KO animal in livestock species, where, in contrast to mice, generation times can be counted by years, rather than by months.

## Mosaicism impairs direct KO generation by CRISPR

**Figure 3:**
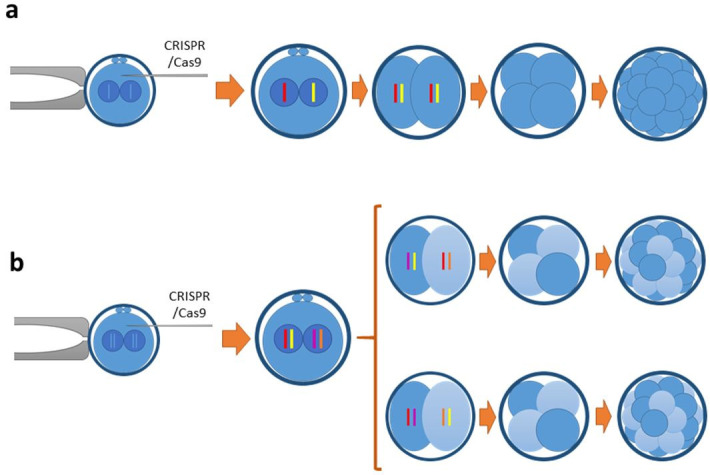
Possible outcomes following CRISPR microinjection into zygotes and NHEJ repair: a) If the DSBs and their definite repairs occur before DNA replication, at the 2n2c stage, 2 indels (alleles) are generated; or b) If DNA has been already replicated (4n4c), CRISPR edition generates 4 alleles that seggregate following first cleavage, leading to two populations of blastomeres harbouring 2 alleles each.

In the context of random generation of indels by NHEJ, a reduction in the number of alleles generated in a given individual is desired to obtain KO individuals: the more alleles an individual harbours, the less probable will be that all of them are frame-disrupting. Ideally, indels should be generated at the 2n2c stage, resulting in 2 alleles. However, DNA replication occurs soon after fertilization in most species and thus genome edition may occur after DNA replication (2n4c), resulting in more than 2 alleles ( Figure 3 ). This is phenomenon is called mosaicism, as it results in mosaic individuals composed by more than one cell population. Mosaicism was initially overlooked, as it is not a common problem in the generation of murine KO models ( Bermejo-Alvarez *et al*., 2015 ), but most of the publications that have performed allele screening following CRISPR direct injection in zygotes have observed mosaicism in different species such as pigs ( Hai *et al*., 2014 ; Sato *et al*., 2015 ; Wang *et al*., 2015c ; Chuang *et al*., 2016 ; Kang *et al*., 2016 ; Petersen *et al*., 2016 ; Yu *et al*., 2016 ; Zhou *et al*., 2016 ; Burkard *et al*., 2017 ; Park *et al*., 2017 ; Whitworth *et al*., 2017 ), goats ( Wang *et al*., 2016a ), sheep ( Crispo *et al*., 2015 ; Wang *et al*., 2016c ; Vilarino *et al*., 2017 ; Zhang *et al*., 2017 ), cattle ( Bevacqua *et al*., 2016 ) and rabbits ( Yan *et al*., 2014 ; Honda *et al*., 2015 ; Guo *et al*., 2016 ; Lv *et al*., 2016 ; Song *et al*., 2016a ; Song *et al*., 2016b ; Sui *et al*., 2016 ; Yang *et al*., 2016 ; Yuan *et al*., 2016 ).

Although it was initially overlooked, the appearance of mosaicism is not surprising given that in most of the cases, conventional IVF or *in vivo* protocols used to collect zygotes for microinjection obtain them at or close to the 2c4n state, which obviously results in at least 4 alleles following edition. In the case of bovine, conventional IVF co-incubates oocytes and spermatozoa for ~20 h ( Parrish *et al*., 1986 ), while DNA replication has been reported to occur between 8 and 18 hours post-insemination ( Eid *et al*., 1994 ). The time of gamete co-incubation used in bovine is roughly similar to those employed in sheep and goats, where pronuclear formation, which precedes DNA replication, occurs even earlier than in bovine ( Mogas *et al*., 1997 ; Gomez *et al*., 1998 ). Pig IVF zygotes are usually obtained after a short 5-6 h gametes co-incubation aiming to reduce polyspermy, whereas according to studies performing sperm injection (ICSI) the onset of S-phase occurs ~10 h after injection ( Kim *et al*., 2003 ). However, pronuclei formation is delayed about 4 h in ICSI-derived embryos ( Kim *et al*., 2003 ) compared to IVF-derived counterparts ( Matas *et al*., 2003 ), and thus porcine zygotes may be at or very close the onset of DNA replication right after IVF. Similarly, *in vivo* porcine zygotes are usually collected at 52-60 hours post-hCG and DNA replication has been reported to occur between 56-60 h post hCG ( Laurincik *et al*., 1995 ). Rabbit zygotes are fertilized at ~14 hours post-mating ( Pincus and Enzmann, 1932 ) and replicate its DNA 3-6 h after sperm penetration ( Oprescu and Thibault, 1965 ; Szollosi, 1966 ). Although it is unclear how long the genome editing mediated by CRISPR combined to the definitive repair of the DSB lasts, it seems that strategies focussed on an earlier delivery of CRISPR components may help to reduce mosaicism.

## CRISPR for KI generation

Targeted insertion of a given sequence can be achieved by homologous recombination ( Orr-Weaver *et al*., 1981 ), which alone (i.e. limited to the incorporation of a sequence containing homology arms) is a very inefficient technique ( Brinster *et al*., 1989 ) that requires the use of an intermediary (ESCs or fibroblasts cultures) to generate a genetically modified animal ( Doetschman *et al*., 1988 ; Schnieke *et al*., 1997 ). However, when a DSB is produced at the HR target locus, the efficiency of HR is improved by >1000 fold ( Moehle *et al*., 2007 ). Under this improved efficiency, the insertion can be directly achieved by co-injecting a HR template and CRISPR components in zygotes, especially when the insert size is small ( Yang *et al*., 2014 ). However, in farm animals the use of fibroblasts as intermediaries followed by SCNT remains being the most commonly used strategy to generate KI animals, as it ensures that all animals generated will carry the intended mutation. For this purpose, the combination of CRISPR+HR template has become the method of choice over HR alone, as the boosted HR efficiency also facilitates genome modification in cell cultures.

The repair template can be double or single-strand DNA (dsDNA or ssDNA). ssDNA often result in higher editing efficiency with reduced random insertions ( Ran *et al.*, 2013b ), but circular vectors are also effective and convenient to introduce long inserts and homology arms ( Yang *et al.*, 2014 ). As previously mentioned, an essential requisite of the HR template to be used combined with CRISPR is that the insertion should disrupt CRISPR recognition site, as otherwise, CRISPR will reproduce the DSB at the reconstituted target site. This can be difficult to achieve when single nucleotide modification is intended, as it can be the case for the introgression of a SNP.

A strategy employed for KI generation is the use of nickase, a mutant form of Cas9 that only produces a break in one strand ( Ran *et al.*, 2013a ). For this purpose, nickase should be co-injected with two sgRNAs (one for each strand), which, in contrast to Cas9, leaves long 5´overhangs that may benefit HR, although not clear consensus has been reached about its putative increased efficiency over conventional Cas9. Another aspect that can be modified from the KO generation protocol is that, as double insertion may be difficult to achieve, the generation of mosaics may be beneficial, as it increases the chances of generating a founder with at least 1 allele harbouring the insertion. Other strategies to improve HR efficiency include the use of NHEJ inhibitors such as SCR-7 ( Singh *et al*., 2015 ) or HR activators as RS-1 ( Song *et al*., 2016a ).

The insertion of a particular sequence at a specific locus allows precise reporter experiments using the endogenous promoter/s and enhancer/s or the endogenously controlled expression of a transgene, among others, but can also be used to generate KO models. For this purpose, a stop codon can be inserted at the beginning of the ORF of a gene. This strategy holds the advantage over conventional KO generation by the random NHEJ-created indels of being easier to genotype, as a restriction enzyme site can be introduced along the stop codon, which allows a sequencing-free identification of the founder offspring. HR can also be used to introduce loxP or FRT sites flanking a target exon for the conditional ablation of genes by Cre-lox ( Orban *et al*., 1992 ) or FLP-FRT ( Buchholz *et al*., 1998 ) recombination systems.

## Applications of CRISPR in livestock research

Genome modification in farm animals holds a myriad of applications on different fields, including the production of therapeutic proteins ( Spencer *et al*., 2005 ; van Veen *et al*., 2012 ; Sheridan, 2016 ), the generation of biomodels for human diseases (reviewed by Whitelaw *et al*., 2016 ), the creation of animal organs less prone to rejection after transplantation (reviewed by Whyte and Prather, 2011 ), the development of human organs generated into an animal host ( Wu *et al*., 2017 ), or, maybe the closest applications to the farm: the improvement of productive rates, animal products, animal health or the environmental impact of farming via genetically modified livestock (reviewed by Lamas-Toranzo *et al*., 2017 ). However, the latter applications are currently stopped by a ban (or lack of approval) of animal products derived from any genetically modified animals (GMAs) for human consumption. Legislation of different countries is slowly adapting to the new scenario created by genome editing (reviewed recently by Van Eenennaam, 2018 ), and the classifications of GMAs into different types depending on the kind of genetic modifications performed could lead to different sets of requirements for approval (discussed in Lamas-Toranzo *et al*., 2017 ). In any case, today CRISPR constitutes a powerful tool for research in livestock species, being readily able to generate knowledge applicable to non-edited livestock.

As it has been previously explained, the benefits of genome modification in research have been largely limited to the mouse model, leaving livestock research devoid of KO or KI models. Although the knowledge generated by some KO or KI murine models can and has been applicable to some aspect of the physiology of livestock species, some processes such as some involved in reproduction, embryo development or infectious disease are highly species specific, impeding the extrapolation of data between species. Besides, CRISPR technology allows to unequivocally prove the role of a particular allele detected on a Genome-wide Association Study (GWAS) on productive traits, which may be helpful when population size or allele frequency is too small to drawn proper conclusions or to test whether such allele will produce a similar phenotype in other genetic background or species. Examples of alleles known to affect production that have been generated by CRISPR include myostatin KOs, which enhance muscular development in CRISPR-edited pigs ( Wang *et al*., 2015a ), goats ( Wang *et al*., 2015b ), sheep ( Crispo *et al*., 2015 ) and rabbits ( Lv *et al*., 2016 ); FGF5 KO in goats ( Wang *et al*., 2016a ), which improves cashmere production; and the POLLED allele introduced in horned bovine genetic lines ( Tan *et al*., 2013 ).

The direct generation of KO by CRISPR is particularly advantageous for its use on experiments aiming to elucidate the molecular aspects of embryo development, as it allows to restrict the ablation from the zygote stage onwards. This contrasts to the approach commonly used in murine KO models, where the low efficiency of HR alone or the lethal phenotype of the homozygous KO force the generation of homozygous KO embryos by the cross of heterozygous (wt/KO) parents ( Evans *et al*., 1985 ). In this context, the gametes originating the KO embryos have been developed in haploinsuficiency (they are wt/KO and then wt or KO as meiosis progresses; Pattabiraman *et al.*, 2015 ), which may lead to confusing conclusions about whether the gene disruption exerted its effect during gametogenesis or during early development. This is especially relevant when the gene of study is involved in stable and long term alterations such as epigenome remodelling ( de Frutos *et al.*, 2016 ). Apart from this advantage, which also applies to the mouse model, the direct generation of a KO embryo circumvents the need of genetically modified animals, as only wt gametes are required to produce KO embryos. Embryonic development in farm animals is known to greatly differ in terms of epigenetic events ( Bermejo-Alvarez *et al*., 2010 ) and early lineage segregation determinants ( Berg *et al*., 2011 ) to the mouse model. Particularly in ungulates, which accounts for the most relevant mammalian livestock species worldwide, the blastocysts does not attach after hatching as it occurs in rodents or humans. Instead, it undergoes a series of developmental events including early and late gastrulation in a period termed embryo elongation. These developmental processes are poorly understood and research on this area is particularly relevant to improve reproductive rates, as failures during this period account for most reproductive losses in pigs ( Bennett and Leymaster, 1989 ) and cattle ( Dunne *et al*., 2000 ; Santos *et al*., 2004 ; Berg *et al*., 2010 ).

Another field that can be greatly benefited from the use of GMA is the research on infectious diseases, especially given the high species specificity of several pathogens. Cattle with increased resistance to tuberculosis have been generated by CRISPR-mediated insertion of natural resistance-associated macrophage protein-1 (*NRAMP1*) ( Gao *et al*., 2017 ). CRISPR has also been used to generate pigs resistant to African Swine Fever by the substitution of the porcine gene RELA for its orthologue from a closely related species that is resistant to the infection: the warthog ( Lillico *et al*., 2016 ). Pigs resistant to the infection of the porcine reproductive and respiratory syndrome virus (PRRVS), a viral disease difficult to eradicate and responsible for major losses in the pig industry, have been generated by CRISPR ( Whitworth *et al*., 2016 ). Although these models were generated thinking about a future use for human consumption, they already provide insights about the pathogenesis and entry ways of infectious agents that can be used to develop therapeutic or prophylactic treatments in conventional non-edited animals.

## Concluding remarks

Genome editing in farm animals has been hampered by the inefficiency and difficulty of early techniques, based on HR combined with SCNT. This obstacle has deprived research in livestock species of the definite answers provided by KO models. The advent of site-specific endonucleases and particularly CRISPR, the easiest to tailor between them, is meant to inaugurate a new era in livestock research. This technology allows direct targeted genome modification in one step by a simple microinjection in zygotes, allowing to unequivocally know the role of a particular gene product on a given process. The novel affordability of KO and KI models for livestock research can improve the quality of scientific results, as it grants the exchange of descriptive and correlational approaches by experimental ones.
